# Defining the minimal clinically important difference for IKDC and KOOS scores for patients undergoing tibial tubercle osteotomy for patellofemoral pain or instability

**DOI:** 10.1002/jeo2.12115

**Published:** 2024-07-29

**Authors:** Julia S. Retzky, Aakash K. Shah, Ava G. Neijna, Morgan E. Rizy, Andreas H. Gomoll, Sabrina M. Strickland

**Affiliations:** ^1^ Hospital for Special Surgery Sports Medicine Institute New York New York USA

**Keywords:** IKDC, KOOS, MCID, TTO

## Abstract

**Purpose:**

The aim of the present study is to define the minimal clinically important difference (MCID) for International Knee Documentation Committee (IKDC) and Knee Injury and Osteoarthritis Outcome Score (KOOS) for patients undergoing tibial tubercle osteotomy (TTO) for either (1) patellofemoral pain or (2) patellar instability.

**Methods:**

Patients undergoing TTO for either patellofemoral pain or patellar instability by one of two sports medicine fellowship‐trained surgeons at a single institution between September 2014 and May 2023 were included in the study. IKDC and KOOS scores were collected preoperatively and minimum 1 year postoperatively. Distribution‐based methods were used to calculate the MCID.

**Results:**

Seventy‐seven patients (82 knees) were included, with a median age of 29.3 years (interquartile range [IQR]: 22.2−36.3 years) and a median BMI of 24.5 kg/m^2^ [IQR: 22.3−28.3 kg/m^2^]. Fifty‐seven patients (74%) were female, and there were 40 right knees (49%). The median time to IKDC and KOOS score was 1.8 and 1.7 years, respectively. Forty‐five patients (46 knees) underwent TTO for patellofemoral instability, and 32 patients (36 knees) underwent TTO for patellofemoral pain. The MCID was 11.5 for IKDC, 10.2 for KOOS pain, 10.1 for KOOS symptoms, 9.9 for KOOS ADL, 14.2 for KOOS sport and 14.2 for KOOS QoL for patients undergoing TTO for patellofemoral pain. The MCID was 11.2 for IKDC, 10.1 for KOOS pain, 10.6 for KOOS symptoms, 10.2 for KOOS ADL, 16.0 for KOOS sport and 13.2 for KOOS QoL for patients undergoing TTO for patellar instability.

**Conclusion:**

We define the MCIDs for commonly used patient‐reported outcome measures for patients undergoing TTO for either patellofemoral pain or patellar instability.

**Level of Evidence:**

Level II.

**Abbreviations**
ADL: activities of daily living
AMZ: anteromedializing
IKDC: International Knee Documentation Committee
IQR: interquartile range
KOOS: Knee Injury and Osteoarthritis Outcome Score
MACI: matrix‐associated autologous chondrocyte implantation
MCID: minimal clinically important difference
MPFLR: medial patellofemoral ligament reconstruction
OCA: osteochondral allograft
PROMs: patient‐reported outcome measures
QoL: quality of life
TTO: tibial tubercle osteotomy
VAS: visual analogue scale

## INTRODUCTION

Patellofemoral pain and instability are common causes of anterior knee pain and disability in adults and children alike [2, 14, 18]. The prevalence of patellofemoral pain is 22.7% and 28.9% in adults and adolescents, respectively [16], and the incidence of patellar instability has been reported to be as high as 29 per 100,000 adolescents [5]. If left untreated, patellar instability renders patients susceptible to chondral defects and ultimately patellofemoral osteoarthritis. The gold standard treatment for recurrent patellar instability with associated patella alta or patellofemoral pain that is refractory to conservative measures includes tibial tubercle osteotomy (TTO). Concomitant medial patellofemoral ligament reconstruction (MPFLR) is often performed for patients with instability.

To assess outcomes following any surgical procedure, including TTO, patient‐reported outcome measures (PROMs), such as International Knee Documentation Committee (IKDC), Knee Injury and Osteoarthritis Outcome Score (KOOS), visual analogue scale (VAS) and Kujala scores, are often collected at both preoperative and postoperative time points. In order to evaluate patient‐perceived meaningful improvements for aforementioned scores, measures such as the minimal clinically important difference (MCID) and the substantial clinical benefit must be defined. The MCID describes the smallest change in a PROM for which a patient perceives clinical benefit [7].

Multiple prior studies have defined the MCID for osteochondral and ligamentous procedures about the knee, including osteochondral allograft (OCA) transplantation [12], MPFLR [17] and anterior cruciate ligament reconstruction [8, 10]. However, there is a paucity of information in the literature regarding MCID values for patients with patellofemoral pain and related procedures. Crossley et al. [3] determined that the MCID for VAS‐U (usual activities) was 1.5−2 points for patients with patellofemoral pain who were managed non‐operatively. Agarwalla et al. [1] found that the MCID for Kujala was 11.9 for patients undergoing TTO for patellofemoral osteoarthritis and pain. The MCID for the KOOS patellofemoral score has been described as 16.4 for patients with patellofemoral pain or osteoarthritis [4], although no study to date has defined the MCID for IKDC or KOOS subscores for patients undergoing TTO for patellofemoral pain. One recent study [13] defined the MCID for IKDC and KOOS subscores for patients undergoing concomitant MPFLR and TTO for recurrent patellar instability.

The aim of the present study is to define the MCID for IKDC and KOOS subscores for patients undergoing TTO for either (1) patellofemoral pain or (2) patellar instability.

## METHODS

### Patient selection

Following institutional review board approval, a retrospective review of a consecutive series of adult patients treated with TTO for patellar instability or patellofemoral pain at a single tertiary care centre between September 2014 and May 2023 was performed. All surgical procedures were performed by one of two senior sports medicine fellowship‐trained orthopaedic surgeons (S. M. S. and A. H. G.).

Patients were included in the study if they underwent TTO for either patellar instability or patellofemoral pain with both preoperative and minimum 1‐year postoperative IKDC and KOOS scores. Patients with incomplete PROM data were excluded from the study.

Patients were categorized into the patellar instability group if they had a history of at least one prior patella dislocation. The remaining patients without a history of patellar dislocation were categorized into the patellofemoral pain group.

Chart review was performed to obtain demographic information as well as information regarding the types of procedures performed.

### Surgical technique and indications

Patients were indicated for TTO if they had either patellar instability or patellofemoral pain with patella alta (Caton−Deschamps index ≥1.3) with a tibial tubercle‐trochlear groove distance ≥15 mm.

A standard anterolateral approach to the tibial tubercle was performed. For an anteromedializing (AMZ) TTO, a 30−45° shingle was cut, leaving a periosteal hinge intact distally. The tibial tubercle was then translated anteromedially 8−10 mm. For a distalizing TTO, a 5 cm wedge‐shaped shingle was cut, a second cut was made at 6 cm and the fragment was moved 8−15 mm distal. An Evans wedge was placed at the proximal aspect of the osteotomy in order to buttress the tibial tubercle. Two 3.5 or 4.5 mm lag screws were placed (lag‐by‐technique), aimed 15° distally.

Postoperatively, patients are made non‐weightbearing in a hinged knee brace locked in extension for 4−6 weeks with a range of motion of 0−30°, increasing as tolerated to 90° by 6 weeks. After 6 weeks, they begin toe touch weightbearing with formal physical therapy for 4−6 months.

### PROMs

IKDC [6] and KOOS [15] were obtained preoperatively and at minimum 1 year postoperatively. For patients who had longer than 1 year follow‐up, IKDC and KOOS scores were completed at the most recent follow‐up visit.

### Calculation of minimal clinically important difference

Distribution‐based methods were used to calculate the MCID. MCID was calculated as 0.5 × the standard deviation of scores based on a previous study [9].

### Statistical analysis

Descriptive statistics including means and standard deviations or frequencies and percentages were used to describe continuous and categorical demographic data, respectively. Baseline demographic information was compared for the patellar instability and patellofemoral pain groups using unpaired *t*‐tests or Mann−Whitney *U* tests for continuous variables based on data distribution per Shapiro−Wilk test results. Categorical variables were analysed with Pearson's chi‐square tests.

Data for change in PROMs for all patients and within groups from preoperative to postoperative were evaluated with paired *t*‐tests or Wilcoxon signed‐rank tests, whereas the change in PROMs between groups from preoperative to postoperative was evaluated with unpaired *t*‐tests or Mann−Whitney *U* tests, as appropriate. Change in PROMs from preoperative to postoperative was compared with paired *t*‐tests or Wilcoxon signed‐rank tests for all patients, patellar instability patients only and patellofemoral pain patients only.

Data analysis was performed with STATA (StataCorp). Statistical significance was determined at the *p* < 0.05 level.

## RESULTS

Of the 100 patients (105 knees) identified, 77 patients (82 knees) met inclusion criteria. Eighteen patients were excluded for incomplete PROM data, and five patients who underwent revision TTO were excluded. Of the patients included in the study, the median age was 29.3 years (interquartile range [IQR]: 22.2−36.3 years), the median BMI was 24.5 kg/m^2^ [IQR: 22.3−28.3 kg/m^2^], the median time to IKDC score was 1.8 years [IQR: 1.1−2.1] and the median time to KOOS was 1.7 years [IQR: 1.1−2.0]. Fifty‐seven patients (74%) were female, and there were 40 right knees (49%). Fifty‐nine patients (72%) had at least one concomitant procedure performed, and 42 patients (51%) had at least one prior surgery. Forty‐five percent of patients had AMZ TTO, 28% had an AMZ and distalizing TTO and 27% had a purely distalizing TTO (Table 1).

**Table 1:** Demographic information for patients included in the study.

		Entire cohort (*n* = 77 patients, 82 knees)	Instability (*n* = 45 patients, 46 knees)	Pain (*n* = 32 patients, 36 knees)	*p* Value
		Mean ± standard deviation or median [interquartile range]			
Age (years)		29.3 [22.2−36.3]	25.3 [20.2−33.5]	33.7 [26.0−38.2]	0.01*
BMI (kg/m^2^)		24.5 [22.3−28.3]	24.6 [22.5−28.3]	25.4 [21.6−28.5]	0.53
Time to IKDC score (years)		1.8 [1.1−2.1]	1.7 [1.1−2.0]	1.9 [1.1−2.1]	0.73
Time to KOOS (years)		1.7 [1.1−2.0]	1.7 [1.1−2.0]	1.9 [1.0−2.0]	0.98
Sex (women)		57 (74)	34 (76)	24 (75)	0.72
Laterality (right)		40 (49)	22 (48)	18 (50)	0.85
Concomitant procedure performed		59 (72)	30 (65)	19 (53)	<0.01*
Previous history of 1+ procedure performed		42 (51)	20 (43)	22 (61)	<0.01*
Type of TTO performed	AMZ	37 (45)	20 (43)	17 (47)	0.56
	distalizing	22 (27)	11 (24)	11 (31)	
	AMZ + distalizing	23 (28)	15 (33)	8 (22)	

Abbreviations: ADL, activities of daily living; AMZ, anteromedializing; IKDC, International Knee Documentation Committee score; KOOS, knee injury and osteoarthritis outcome score; QoL, quality of life; TTO, tibial tubercle osteotomy.

* Statistical significance at *p* < 0.05 level.

Of the 77 patients included in the study, 45 patients (46 knees) underwent TTO for patellofemoral instability, and 32 patients (36 knees) underwent TTO for patellofemoral pain. The patellofemoral pain group was older than the instability group (median age 33.7 years [IQR: 26.0–38.2 years] vs. 25.3 years [IQR: 20.2−33.5 years], respectively, *p* = 0.01), and the instability group was more likely than the pain group to have one or more concomitant procedures (*p* < 0.01). The pain group, however, was more likely to have a history of one or more previous procedures relative to the instability group (*p* < 0.01). There were otherwise no differences between groups with respect to BMI, time to IKDC, time to KOOS, sex, laterality and type of TTO performed (*p* > 0.05 for all, Table 1). The average distalization distance was 9.1 ± 1.0 mm, and the average anteromedialization distance was 9.5 ± 2.3 mm. There were no differences between groups with respect to the amount of distalization or anteromedialization performed (*p* = 0.53).

The most common concomitant procedure performed was MPFLR (30 knees, 37%), followed by matrix‐associated autologous chondrocyte implantation of the patella (23 knees, 28%). There were no differences between groups with respect to the types of concomitant procedures performed (*p* > 0.05 for all, Table 2).

**Table 2:** Concomitant procedures were performed for the entire cohort and the pain and instability groups.

	Entire cohort (*n* = 77 patients, 82 knees)	Instability (*n* = 45 patients, 46 knees)	Pain (*n* = 32 patients, 36 knees)	*p* Value
MPFLR	30 (37)	28 (61)	2 (6)	0.10
MACI patella	23 (28)	11 (24)	12 (33)	0.08
Lateral release	19 (23)	14 (30)	5 (14)	0.91
Chondroplasty patella	17 (21)	11 (24)	6 (17)	0.93
OCA patella	15 (18)	8 (17)	7 (19)	0.56

*Note*: Statistical significance at *p* < 0.05 level.

Abbreviations: MACI, matrix‐associated autologous chondrocyte implantation; MPFLR, medial patellofemoral ligament reconstruction; OCA, osteochondral allograft.

There was a significant increase in IKDC and all KOOS subscores from preoperative to minimum 1 year postoperative (*p* < 0.01 for all, Table 3).

**Table 3:** The change in IKDC and KOOS subscores from preoperative to minimum 1 year postoperative for the entire cohort.

	Preoperative score	Postoperative score	Delta	*p* Value
	Mean ± standard deviation or median [interquartile range]			
IKDC	43.4 ± 16.0	69.7 ± 19.5	26.4 ± 22.1	<0.01*
KOOS pain	63.3 ± 16.7	82.9 ± 15.1	19.5 ± 18.3	<0.01*
KOOS symptoms	59.7 ± 17.9	80.5 ± 14.8	20.8 ± 20.1	<0.01*
KOOS ADL	75 [60.3−89.7]	97.1 [83.8−100.0]	10.3 [4.4−26.5]	<0.01*
KOOS sport	30.0 [10.0−45.0]	65.0 [40.0−85.0]	30.0 [5.0−45.0]	<0.01*
KOOS QoL	25.1 ± 16.2	58.3 ± 25.6	33.3 ± 26.6	<0.01*

Abbreviations: ADL, activities of daily living; IKDC, International Knee Documentation Committee score; KOOS, knee injury and osteoarthritis outcome score; QoL, quality of life; TTO, tibial tubercle osteotomy.

* Statistical significance at *p* < 0.05 level.

The median preoperative KOOS ADL score was higher in the instability group (79.41 [IQR: 67.7−89.7] compared to the pain group (70.6 [IQR: 52.9−84.6], *p* = 0.047); otherwise, there were no differences between the instability and pain groups with respect to preoperative or postoperative IKDC or KOOS subscores (*p* > 0.05 for all). There were no differences between the pain and instability groups with respect to the change in IKDC and all KOOS subscores from preoperative to minimum 1 year postoperative (*p* > 0.05 for all, Table 4).

**Table 4:** The change in IKDC and KOOS subscores from preoperative to a minimum 1 year postoperative for the instability and pain cohorts.

	Instability (*n* = 45 patients, 46 knees)	Pain (*n* = 32 patients, 36 knees)	*p* Value
	Mean ± standard deviation or median [interquartile range]		
IKDC	26.3 ± 23.7	26.4 ± 21.0	0.99
KOOS pain	16.7 [2.8−27.8]	23.6 [3.6−38.9]	0.33
KOOS symptoms	16.1 [7.1−35.7]	23.2 [0.01−30.4]	0.94
KOOS ADL	10.3 [2.9−22.1]	15.4 [5.9−41.9]	0.23
KOOS sport	30.0 [15.0−40.0]	30.0 [2.5−47.5]	0.83
KOOS QoL	31.3 [18.8−50.0]	34.3 [9.4−50.0]	0.78

*Note*: Statistical significance at *p* < 0.05 level.

Abbreviations: ADL, activities of daily living; IKDC, International Knee Documentation Committee score; KOOS, knee injury and osteoarthritis outcome score; QoL, quality of life; TTO, tibial tubercle osteotomy.

The MCID for each PROM for the instability cohort was as follows: 11.2 for IKDC, 10.1 for KOOS pain, 10.6 for KOOS symptoms, 10.2 for KOOS ADL, 16.0 for KOOS sport and 13.2 for KOOS QoL. The MCID for each PROM for the pain cohort was as follows: 11.5 for IKDC, 10.2 for KOOS pain, 10.1 for KOOS symptoms, 9.9 for KOOS ADL, 14.2 for KOOS sport and 14.2 for KOOS QoL (Table 5).

**Table 5:** The MCID for IKDC and KOOS subscores for patients undergoing TTO for either patellofemoral pain or instability at minimum 1 year postoperative.

	Instability (*n* = 45 patients, 46 knees)	Pain (*n* = 32 patients, 36 knees)
IKDC	11.2	11.5
KOOS pain	10.1	10.2
KOOS symptoms	10.6	10.1
KOOS ADL	10.2	9.9
KOOS sport	16.0	14.2
KOOS QoL	13.2	14.2

Abbreviations: ADL, activities of daily living; IKDC, International Knee Documentation Committee score; KOOS, knee injury and osteoarthritis outcome score; MCID, minimal clinically important difference; QoL, quality of life; TTO, tibial tubercle osteotomy.

## DISCUSSION

We found that the MCID was 11.5 for IKDC, 10.2 for KOOS pain, 10.1 for KOOS symptoms, 9.9 for KOOS ADL, 14.2 for KOOS sport and 14.2 for KOOS QoL for patients undergoing TTO for patellofemoral pain. The MCID was 11.2 for IKDC, 10.1 for KOOS pain, 10.6 for KOOS symptoms, 10.2 for KOOS ADL, 16.0 for KOOS sport and 13.2 for KOOS QoL for patients undergoing TTO for patellar instability.

Our results are in line with Qiao et al. [13], who defined the MCID as 9.9 for IKDC, 9.0 for KOOS pain, 10.8 for KOOS symptoms, 10.0 for KOOS ADL, 17.8 for KOOS sport and 12.7 for KOOS QoL. The present study builds on the significantly scarce body of literature by defining the MCID for commonly used PROMs for patients undergoing TTO for patellofemoral pain.

There are several limitations to the present study. First, 82% of patients had concomitant procedures, such as OCA transplantation or autologous chondrocyte implantation, which may limit the generalizability of our results. However, the majority of patients who undergo TTO have concomitant chondral pathology; so, we feel that our sample accurately reflects the population of patients who undergo this procedure. Moreover, our sample size is relatively small, although the sample size used in the present study is similar to other studies that have defined the MCID for common orthopaedic procedures, such as OCA transplantation [12] and autologous chondrocyte implantation [11].

In sum, we describe the MCIDs of several commonly used PROMs for patients undergoing TTO for patellofemoral pain or instability. Identifying the minimal change in PROMs necessary to achieve clinical improvement is important for understanding and improving outcomes for patients undergoing TTO.

## AUTHOR CONTRIBUTIONS

Julia S. Retzky performed data analysis and writing of the manuscript. Aakash K. Shah performed data collection and editing of the manuscript. Ava G. Neijna performed data collection and editing of the manuscript. Morgan E. Rizy performed data collection and editing of the manuscript. Andreas H. Gomoll conceived of the study and was involved in manuscript editing. Sabrina M. Strickland conceived of the study and was involved in manuscript editing. All authors read and approved the final manuscript.

## CONFLICTS OF INTEREST STATEMENT

Andreas H. Gomoll reports consulting or speaking fees for Bioventus, LLC; CartiHeal Inc.; JRF Ortho; and LinvatecCorp.; Miach Orthopaedics Inc.; Moximed Inc.; Organogenesis Inc.; Pacira Pharmaceuticals Inc.; Smith & Nephew Inc.; and Vericel Corp.; and equity or stocks in Engage Surgical; Smith & Nephew Inc.; and Stryker. Sabrina M. Strickland reports consulting or speaking fees for Miach Orthopaedics Inc.; Moximed Inc.; Smith & Nephew Inc.; and Vericel Corp.; and equity or stocks in Engage Surgical; Smith & Nephew Inc.; and Stryker. The remaining authors declare no conflict of interest.

## ETHICS STATEMENT

Ethical approval was approved by the Hospital for Special Surgery Institutional Review Board. IRB approved, #2020‐2123. Informed consent is obtained prior to enrolment in the study.

## Data Availability

Data are available on request from the authors.
